# Effect of Growth Regulators on *Stevia rebaudiana* Bertoni Callus Genesis and Influence of Auxin and Proline to Steviol Glycosides, Phenols, Flavonoids Accumulation, and Antioxidant Activity In Vitro

**DOI:** 10.3390/molecules25122759

**Published:** 2020-06-15

**Authors:** Aušra Blinstrubienė, Natalija Burbulis, Neringa Juškevičiūtė, Nijolė Vaitkevičienė, Rasa Žūkienė

**Affiliations:** Institute of Biology and Plant Biotechnology, Agriculture Academy, Vytautas Magnus University, Donelaicio Str. 58, 44248 Kaunas, Lithuania; ausra.blinstrubiene@vdu.lt (A.B.); natalija.burbulis@vdu.lt (N.B.); nijole.vaitkeviciene@vdu.lt (N.V.); rasa.zukiene@vdu.lt (R.Ž.)

**Keywords:** antioxidant activity, callus, flavonoids, *Stevia rebaudiana* Bertoni, phenolic compounds, rebaudioside A, stevioside

## Abstract

Stevia is a plant containing many active compounds, but usually propagated by stem cuttings because of low seed-yield-germination ability. The aim of this study was to investigate the impact of plant-growth regulators on stevia callus induction and growth from somatic tissue, as well as to determine the effect α-naphthalene acetic acid (NAA) and proline (PRO) on the amount of stevioside, rebaudioside A, phenols, flavonoids, and antioxidant activity. Stem and leaf segments were inoculated on a Murashige and Skoog (MS) medium supplemented with different concentrations of NAA and 6-benzylaminopurine (BAP) for callus genesis. The amount of steviol glycosides (SGs) was evaluated using high-performance liquid chromatography (HPLC), and the amounts of total phenols, flavonoids, and antioxidant activity by spectrophotometric methods. The highest callus-induction frequency and callus-mass increase were obtained from the leaf explants in MS medium supplemented with 2.0 μM NAA. The highest amount of SGs, phenols, and flavonoids, and stronger antioxidant activity were determined in the cellular compounds of callus from leaf explant. PRO reduced the amount of SGs and flavonoids. The significantly highest amount of total phenolic compounds was obtained in the callus from leaf explants in the medium supplemented with 2.0 µM NAA and 2.0 µM PRO.

## 1. Introduction

*Stevia rebaudiana* Bertoni is a herbaceous perennial plant of the *Asteraceae* family [1]. The natural habitat of stevia extends from the southwestern United States to the Brazilian highlands [2]. The stevia genus comprises about 300 species [3].

Plants are the most important source of biologically active compounds [4]. Nowadays, stevia is globally one of the most studied plants because its leaves are used as a sweetener in Japan, Korea, China, and South America [5]. *Stevia rebaudiana* Bertoni is a perennial plant with many active compounds, especially steviol glycosides (SGs) that are present mainly in the leaves and other parts of the plant [6]. Eight ent-kaurene glycosides, namely dulcoside A, rebaudiosides A–E, steviolbioside, and stevioside, are the main compounds of the diterpene derivative steviol [7,8]. The leaves of these plants contain 0.3% dulcoside, 0.6% rebaudioside C, 3.8% rebaudioside A, and 9.1% stevioside [3]. Very high sweetness is characteristic to SGs, i.e., about 300 times sweeter than sucrose, without any generated energy and a glycemic index of zero [9,10]. In addition to the SGs, this plant contains a high amount of phenolic compounds and flavonoids, and has high antioxidant activity [4,11]. Flavonoids and phenolic acids are the most important groups of secondary metabolites and bioactive compounds in plants [12]. Phenolic compounds are secondary metabolites that are derived from the pentose phosphate, shikimate, and phenylpropanoid pathways in plants [13]. These compounds play an important role in growth and reproduction, providing protection against pathogens and predators [14]. They are also natural products and antioxidant substances capable of scavenging free superoxide radicals, antiaging, and reducing the risk of cancer [15]. Researchers determined that stevia extracts contain significant amounts of biologically active phytochemicals with antioxidant activity that could be used in industry, for example, as food and cosmetics ingredients or dietary supplements [16]. Stevia is useful in many areas: to undergo treatment for cavities, depression, diabetes, fatigue, heart support, hypertension, hyperglycemic, infections, obesity, sweet cravings, tonic, urinary insufficiencies, and as a sweetener [17]. The propagation of stevia by seeds and cutting is problematic because the germination of this plant’s seeds is very low [18], and the amount of cuttings from one plant is limited, so researchers are looking for alternative stevia-propagation methods.

Plant growth and development can be controlled by exogenous and endogenous growth regulators, such as hormones [19,20]. Auxins, abscisic acid, cytokinins, ethylene, and gibberellins are commonly known as the main natural plant hormones. Auxins, cytokinins, and auxin–cytokinin interactions are usually considered to be the most important for regulating the growth process and development of plant tissue and organ cultures [20,21,22]. Exogenously applied plant-growth regulators are biologically active and equal to the equivalent endogenous hormones, or even exceed their activity. In addition to these compounds, many chemicals that interfere with and inhibit the synthesis, transport, or action of endogenous hormones are available. Plant-growth regulators are factors that help to study the role of plant hormones in in vitro cultures [23].

Nowadays, extensive research has been done on the accumulation of secondary metabolites in plants affected by abiotic/biotic stress [24]. Plant productivity and secondary-metabolite biosynthesis are affected by abiotic factors such as alkalinity or low pH, which could also be changed by proteinogenic amino acid proline (PRO) [25,26]. SGs are actively studied in stevia plants. Researchers reported different results on SG accumulation in callus culture. In the callus tissue of stevia from leaf explants after 70 days of cultivation, the stevioside amount was 16.24% of callus dry mass, and it was two and four times higher than that in the leaf and flowers of the same plant, respectively [27]. However, other authors did not find any presence of stevioside in the callus in vitro [28]. Other authors found that additional factors, such as proline and polyethylene glycol, may influence processes in the plants and the amount of SGs. Proline is a proteinogenic amino acid for primary metabolism [29,30]. However, proline is usually considered to be a metabolite with protective functions, but the exogenous proline can be also deleterious to plants and inhibit growth and cell division [31]. As far as we know, studies about the effect of auxin and proline combinations to the amount of phenols, flavonoids, and antioxidant activity in stevia callus from leaf and stem have not yet been implemented.

Therefore, the aim of this study was to investigate the impact of plant-growth regulators on stevia callus induction and growth from somatic tissue, as well as to determine the effect that α-naphthalene acetic acid and proline have on the amount of stevioside, rebaudioside A, phenolic compounds, flavonoids, and antioxidant activity of cellular compounds of callus.

## 2. Results and Discussion

### 2.1. Callus Induction of Stevia rebaudiana

In this study, callus induction from stem and leaf segments of *S. rebaudiana* Bertoni was explored on Murashige and Skoog (MS) medium without growth regulators, with auxin, α-naphthalene acetic acid (NAA; 1.0–3.0 µM), and with or without cytokinin 6-benzylaminopurine (BAP; 1.0 µM) (Table 1).

The highest callus-formation frequency from leaf explants after one week was in the medium supplemented with 2.0 µM NAA compared with the control. In this treatment, callus frequency was 83.3%. Callus-formation frequency from stem segments in the medium with 3.0 µM NAA was basically highest compared with the results of the medium with 1.0 or 2.0 µM NAA; the average was 63.8%.

Callus-induction frequency was also determined after two weeks. Callus-culture-induction frequency from leaf explants was higher in comparison with the results from stem segments (on average, 97.95% and 80.85%, respectively). In MS media with 1.0 NAA µM, 2.0 NAA µM, 1.0 µM NAA + 1.0 µM BAP, 2.0 μM NAA + 1.0 µM BAP, and 3.0 µM NAA + 1.0 µM BAP, callus-formation frequency from the leaf was 100%. Of the stem segments, 91.0% formed a callus culture in the MS medium with 2.0 μM NAA. After two weeks in each research treatment, callus-induction frequency from the leaf explants was higher; therefore, this explant type was more appropriate for *Stevia rebaudiana* Bertoni callus induction.

Stevia vegetative propagation through cuttings is also limited because of the low rate of multiplication and growth [17]; thus, scientists are still searching for propagation alternatives. Callus induction is one of the ways to obtain identical plants. Although in our study we used BAP and NAA, other researchers use kinetin (KIN), indole-3-acetic acid (IAA), 2,4-dichlorophenoxyacetic (2,4-D), or amino acids, as well as BAP and NAA [17,32,33].

Other authors showed different results compared to ours and their callus induction; their callus induction was determined after four weeks (twice as long). For callus induction from leaf explants, they used the same concentration of NAA and BAP (1.0 µM NAA + 1.0 µM BAP and 2.0 µM NAA + 1.0 µM BAP), but callus induction was 60.0% ± 4.71% (1.0 µM NAA + 1.0 µM BAP) and 52.0% ± 6.32% (2.0 µM NAA + 1.0 µM BAP). These authors achieved 100% callus induction using 1.0 µM NAA + 1.0 µM 2,4-D [34]. The other researcher groups reported that explants in MS media with 2,4-D or BAP (2.0–3.0 mg L^−1^) and NAA 2.0 mg L^−1^ formed maximal callus after two [35,36] and three weeks [37]. Patel and Shah (2009) [37] reported that callus induction from leaf explant was 72.5% on MS media with 2.0 µM NAA + 1.0 µM BAP after three weeks. In the present study, 100% callus-formation frequency from leaf explants in the same medium was obtained after two weeks.

### 2.2. S. rebaudiana Callus-Mass Increase

The composition of the medium plays an important role in determining the morphogenetic pathway [38]; therefore, it is an important parameter for callus development and growing control. The callus culture was transferred to the same composition media, and callus-mass increase was evaluated after one and two months of cultivation (Table 2).

**Figure 1:**
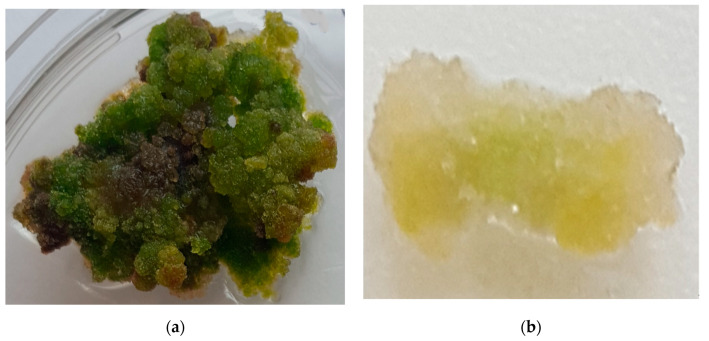
Callus from (**a**) leaf (bar = 2.0 mm) and (**b**) stem segment (bar = 1.0 mm) explants.

After one month, the mass of the callus from leaf explants (Figure 1a) was seven times higher than the callus mass from the stem segments (Figure 1b) in the MS medium with 2.0 µM NAA. The cytokinin BAP and NAA combination had a positive effect on the growth of callus mass compared with that of the control. When we compared the effect of NAA alone with the combination of NAA and BAP, we determined that BAP had a significant positive effect on mass. Average callus mass from leaf explants in all media with NAA (1.0–3.0 µM) was 15.03 mg, but in the media supplemented with NAA and BAP (1.0 µM NAA + 1.0 µM BAP, 2.0 µM NAA + 1.0 µM BAP, 3.0 µM NAA + 1.0 µM BAP) average mass was 16.77 mg, i.e., significantly higher.

After two months of cultivation, the highest mass of callus from leaf and stem segments was in the medium with 2.0 µM NAA, compared with other treatments. the results show that BAP only had a positive effect on the mass of callus from leaf explants because, after two months, callus mass was higher in the medium with 1.0 µM NAA + 1.0 µM BAP compared with the results of the medium with only 1.0 µM NAA.

Researchers determined that the callus mass on the MS medium supplemented with 1.0 µM NAA + 0.5 µM BAP was 83.56 mg (from leaf explants after six weeks) [39]. They used twice lower concentration of BAP compared with ours. Our results show that callus mass on the MS medium with 1.0 µM NAA + 1.0 µM BAP was 26.5 mg, thus a twice higher concentration of BAP could reduce callus-mass growth. 

In many cases, callus mass from the leaf explants was higher in comparison to that from the stem segments, so the leaf explant is more suitable for obtaining a higher callus mass of *Stevia rebaudiana* Bertoni. Culture medium supplemented with 2.0 µM NAA was the most appropriate for mass increasing of the callus developed from the leaf explant.

### 2.3. NAA and PRO Effect on SG Accumulation in S. rebaudiana Callus

In this study, SG accumulation in callus culture from stem and leaf segments of *S. rebaudiana* Bertoni was explored on the MS medium with auxin 2.0 µM NAA, and with the NAA combination with PRO (2.0–5.0 µM). 

After HPLC analyses, we determined that the amount of stevioside was significantly higher from the leaf-explant-formed callus compared with that of the stem segments (an average of the leaf explants was 0.33 mg g^−1^, from stem segments, it was 0.04 mg g^−1^; Table 3); the amount of stevioside from the leaf callus was eight times higher. In MS media with 2.0 µM NAA and 2.0 µM NAA + 4.0 µM PRO, the amount of stevioside was significantly higher compared with that of the other treatment. 

The amount of rebaudioside A was lower in all cases compared with stevioside content except for one medium—2.0 µM NAA + 5.0 µM PRO. In this medium, the callus from the stem segments contained the same amount of stevioside and rebaudioside A. In the medium with 2.0 µM NAA, the content of rebaudioside A from the leaf explant was significantly higher compared with other treatments.

In the callus culture from the leaf explants, the content of stevioside and rebaudioside A was significantly higher compared with the results of callus from stem segments. Furthermore, we determined basically the same, that is, that PRO reduced the SG content. In all treatments, the amount of stevioside was higher in comparison with rebaudioside A.

However, PRO may be useful for altering the SG ratio in stevia callus. This biologically active substance only changed the RebA/Stev ratio in the callus from the leaf explants only. The significantly highest ratio of the RebA/Stev was obtained in the callus from the stem segments cultured on the medium with 2.0 µM NAA + 5.0 µM PRO. Studies of other authors showed that abiotic stress in plants, for example, the presence of higher concentrations of PRO to the culture medium, induced various metabolic changes in plant-cell culture [40].

In plant-tissue culture, both biotic and abiotic factors can be used to induce secondary metabolite biosynthesis in plant cells [30]. Growth regulators are the factor that can change the amount of rebaudioside A and stevioside, and the SG ratio. It is especially important when increasing the RebA/Stev ratio in order to reduce the bitter aftertaste that is characteristic to powdered stevia [41,42]. RebA is superior in terms of both sweetness and quality of taste, whereas stevioside is usually perceived to have a significant bitter aftertaste [43]. Stevioside has an undesirable bitter taste. It is possible that reducing the amount of this compound or increasing the Steve/RebA ratio will also reduce bitter taste.

Swanson et al. (1992) [28] showed that callus cultures or buds did not contain stevioside. They described that these compounds can only be found in fully developed plants. However, Hsing et al. (1983) [27] and Taware et al. (2010) [44] determined double the amount of SGs throughout the plant than that in the leaves or found high content of stevioside in the callus cultures.

### 2.4. NAA and PRO Effect on Accumulation of Total Phenolic Compounds and Total Flavonoids in S. rebaudiana Callus

**Figure 2:**
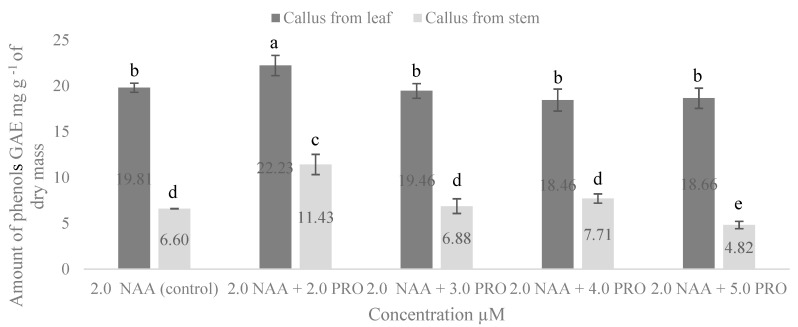
Effect of NAA and PRO concentration on accumulation of total phenolic compounds in callus formed from stem and leaf explants. Means not sharing a common letter were significantly different (*p* < 0.05). NAA, α–naphthalene acetic acid; PRO, proline.

In this study, we determined the amount of phenolic compounds in callus culture formed on media with 2.0 µM NAA and NAA combined with various concentrations of PRO (Figure 2). The medium with 2.0 µM NAA was considered as the control, because, on the medium without cytokinin, the callus was not observed. Analysis of the total phenolic compounds showed that the amount of phenols in the callus from the leaf explants was higher than that from stem segments; however, PRO only had a positive effect in the medium with 2.0 µM NAA + 2.0 µM PRO. The significantly highest amount of phenolic compounds was obtained in the callus formed from leaf explants in the medium supplemented with 2.0 µM NAA + 2.0 µM PRO compared with the other treatments. In the medium supplemented with 2.0 µM NAA + 2.0 µM PRO, phenolic compounds in the callus formed from the stem segments significantly increased by 1.7 times compared with the control.

**Figure 3:**
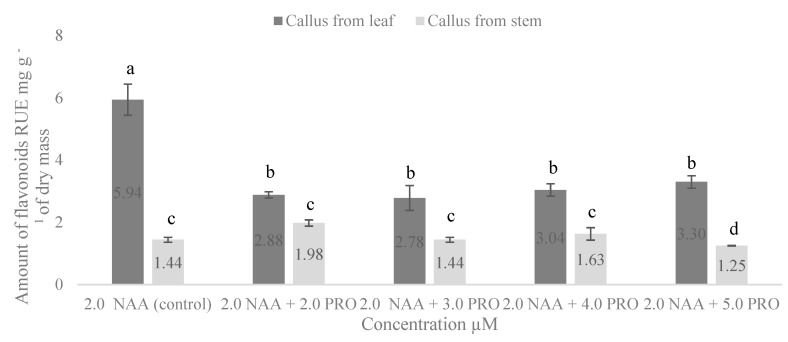
Effect of NAA and PRO concentration on accumulation of total flavonoids in callus culture from stem and leaf explants. Means not sharing a common letter were significantly different (*p* < 0.05). NAA, α–naphthalene acetic acid; PRO, proline.

The dependence of flavonoid amount on callus origin appeared to basically be similar to that for the phenolic compounds. A higher amount of flavonoids was found in the callus formed from the leaf segments (Figure 3). The significantly highest amount (5.94 mg g^−1^) of flavonoids was obtained in the callus formed from the leaf segments in the medium supplemented with only 2.0 µM NAA. Proline significantly reduced the amount of flavonoids in the callus from the leaf explants in each treatment. The study revealed an increase of flavonoids in the callus from the stem segments on the medium supplemented with 2.0 µM NAA + 2.0 µM PRO and 2.0 µM NAA + 4.0 µM PRO; however, differences were not significant.

Phenolic compounds are commonly found in both edible and nonedible plants, and they were reported to have multiple biological effects, including antioxidant activity [45]. Researchers identified 18 phenolic compounds that demonstrated the high antioxidant capacity of the stevia leaf [46]. High levels of total phenols and flavonoids were also found in stevia infusions [47]. Our study showed that the amount of phenolic compounds and flavonoids in all cases was higher in the callus from the leaf explants. Stevia leaves are increasingly consumed as infusions because of their antioxidant properties, and stems are valuable for the high levels of flavonoids and phenolic compounds [48]. Our results reveal that the amount of phenolic compounds varied from 4.82 to 11.43 mg g^−1^ in the stevia callus from the stem segments, and from 18.46 to 22.23 mg g^−1^ in the callus from the leaf explants; the amount of flavonoids was 1.25–1.98 mg g^−1^ in the callus from the stem segments, and 2.78–5.94 mg g^−1^ in the callus from the leaf explants. Kwok and Shetty (1997) [49] investigated *Thymus vulgaris* L. and determined that proline oxidation via proline dehydrogenase may not be operating efficiently; therefore, the pentose phosphate pathway and thus phenolic synthesis may not be stimulated. This could be a reason for the decline of phenolic compounds in callus culture that was influenced by proline. However, to the best of our knowledge, there is no other published research on the effects of auxin and proline combinations on the amount of total phenols and flavonoids in stevia callus from leaf and stem explants.

### 2.5. NAA and PRO Effect on Antioxidant Activity in S. rebaudiana of Cellular Compounds of Callus

**Figure 4:**
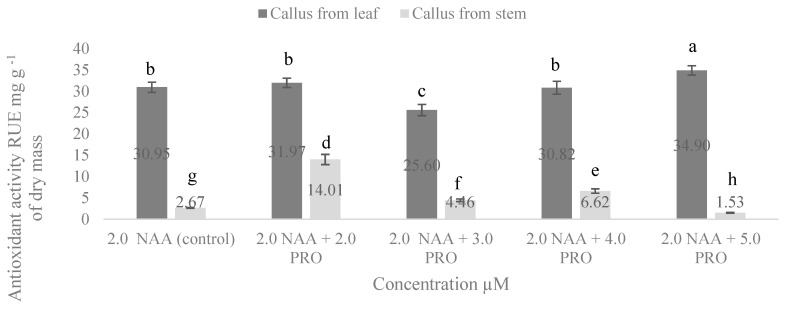
Effect of NAA and PRO concentration on antioxidant activity in cellular compounds of callus culture from stem and leaf explants. Means not sharing a common letter were significantly different (*p* < 0.05). NAA, α–naphthalene acetic acid; PRO, proline.

Antioxidant activity of cellular compounds of callus from stem and leaf explants is presented in Figure 4. Our study showed that antioxidant activity in all cases was higher in the cellular compounds of callus from the leaf explants. The highest antioxidant activity in the cellular compounds of callus from the stem segments was in the medium with 2.0 µM NAA + 2.0 µM PRO. In media supplemented with 2.0 µM NAA + 3.0 µM PRO and 2.0 µM NAA + 4.0 µM PRO, antioxidant activity in the cellular compounds of callus from the stem segments significantly also increased compared with the control. In the cases of the cellular compounds of callus from the leaf explants, antioxidant activity was significantly increased when exposed to 2.0 µM NAA + 5.0 µM PRO, while 2.0 µM NAA + 3.0 µM PRO in the MS medium significantly decreased it compared with the control treatment.

According to Tadhani et al. (2007) [4], stevia callus has strong antioxidant activity. They found that the total antioxidant activity ranged from 9.44 to 37.36 mg g^−1^ and from 10.14 to 34.37 mg g^−1^ depending on different standards in the water, and the methanolic extract of stevia callus from the leaf, respectively.

Our study evidenced that the level of correlation between the content of total phenolics and antioxidant activity in *S. rebaudiana* Bertoni cellular compounds of callus from leaf and stem explants depended on the applied concentrations of proline (Table 4). The strongest positive correlation between total phenolics and antioxidant activity was found in the cellular compounds of callus from the leaf explants treated with 2.0 µM NAA + 3.0 µM PRO compared to the control. However, in the cases of the callus from the stem segments, all proline treatments gave less correlation compared to the control. Samadi et al. (2019) [50] found a positive correlation between antioxidant activities and total phenol in stevia-plant callus. According to these authors, phenolic compounds may play a key role in the antioxidant activity of stevia. Significant correlations between *S. rebaudiana* callus antioxidant activity and the content of total phenolics were found in another study as well [51].

### 2.6. Principal-Component Analysis (PCA)

**Figure 5:**
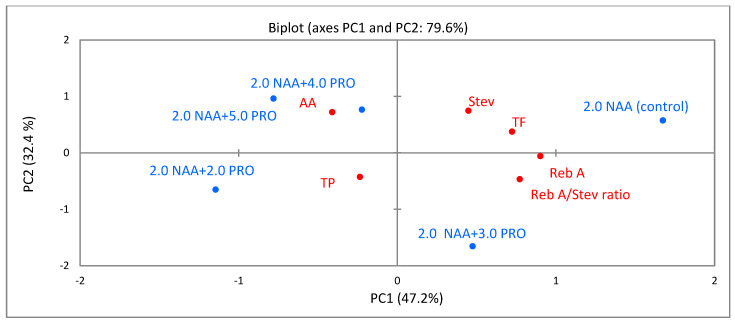
Principal-component analysis **(**PCA) for total phenolics (TP), total flavonoids (TF), stevioside (Stev), rebaudioside A (Reb A), rebaudioside A/stevioside ratio, and antioxidant activity (AA) in cellular compounds of callus from leaf explants influenced by different concentrations and combination of α-naphthalene acetic acid (NAA) and proline (PRO) (2.0 µM NAA (control), 2.0 µM NAA + 2.0 µM PRO, 2.0 µM NAA + 3.0 µM PRO, 2.0 µM NAA + 4.0 µM PRO, and 2.0 µM NAA + 5.0 µM PRO).

**Figure 6:**
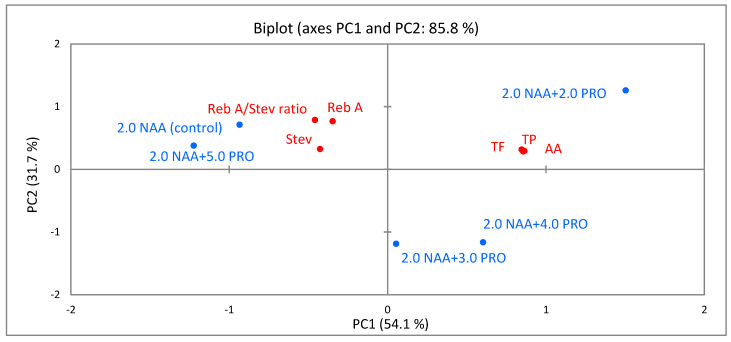
PCA for total phenolics (TP), total flavonoids (TF), stevioside (Stev), rebaudioside A (Reb A), rebaudioside A/stevioside ratio (reb A/Stev ratio), and antioxidant activity (AA) in cellular compounds of callus from stem explants influenced by different combinations and concentrations of α-naphthalene acetic acid (NAA) and proline (PRO) (2.0 µM NAA (control), 2.0 µM NAA 2.0 + µM PRO, 2.0 µM NAA + 3.0 µM PRO, 2.0 µM NAA + 4.0 µM PRO and 2.0 µM NAA + 5.0 µM PRO).

PCA was carried out to evaluate associations between different applied concentrations of proline and the identified bioactive compounds, ratio of rebaudioside A/stevioside, and the antioxidant activity in *S. rebaudiana* Bertoni cellular compounds of callus from leaf (Figure 5) and stem (Figure 6) explants.

As shown in Figure 5, the first principal component (PC1) explained 47.2% of variance, and the second principal component (PC2) explained 32.4% of the variance, representing 79.6% of total variance. PC1 was associated with total flavonoids, rebaudioside A, and the ratio of rebaudioside A/stevioside. PC2 was characterized by steviosides and antioxidant activity. The biplot showed that the identified bioactive compounds, rebaudioside A/stevioside ratio, and antioxidant activity of the cellular compounds of callus from the leaf explants differed with different concentrations of proline because all treatments were well-separated in the PCA map.

As shown in Figure 6, the first two axes (PC1 and PC2) describe 85.8% of the total variation. PC1 accounted for 54.1% of variation in the data and was strongly positively associated with total phenolics, total flavonoids, and antioxidant activity.

PC2 accounted for 31.7% of the total variation and was positively correlated with rebaudioside A/stevioside ratio and rebaudioside A. PCA results show that the higher content of total phenolics and total flavonoids and stronger antioxidant activity in the cellular compounds of callus from the stem segments ertr positively associated with the application of 2.0 µM NAA + 2.0 µM PRO. The higher content of stevioside and rebaudioside A and their higher ratio were associated with the application of 2.0 µM NAA + 5.0 µM PRO and the control treatment (without the addition of proline).

## 3. Materials and Methods

### 3.1. Plant Material

This study was carried out during 2018–2020 at the Laboratory of Agrobiotechnology of the Institute of Biology and Plant Biotechnology, and the Department of Biochemistry of Vytautas Magnus University. To sterilize the seeds, they were soaked in 70% ethanol for 30 s and 0.1% natrium hypochloride for 20 s, respectively, followed by an extensive wash with sterile water for a couple of times. Sterilized seeds were grown in vitro on basal MS medium [52] without growth regulators, supplemented with 10.0 g L^−1^ sucrose and 8.0 g L^−1^ agar (Carl Rith GmbH, Germany). The seeds were placed into Petri dishes with media (20 mL), and the cultures were incubated at a temperature of 27 ± 2 °C, under illumination of 50 μmol m^−2^ s^−1^ and a photoperiod of 16/8 h (day/night). Explant transfer of the culture was carried out under aseptic conditions.

### 3.2. In Vitro Preparation of S. rebaudiana Material

In the current study, two different explants, i.e., leaf and stem segments, taken from 6-week-old in vitro grown stevia plants, were selected for callus induction. In this study, we investigated the effect of different concentrations and combinations of auxin and cytokinin on callus induction and growth. For this purpose, explants were cultured in MS medium supplemented with different (1.0–3.0 µM) concentrations of NAA and combination of NAA and BAP (1.0 µM NAA + 1.0 µM BAP; 2.0 µM NAA + 1.0 µM BAP, and 3.0 µM NAA + 1.0 µM BAP) as well as 30.0 g L^−1^ sucrose and 8.0 g L^−1^ agar. The results of study of callus induction and callus mass increases showed that the best results were obtained in medium with 2.0 µM NAA, so further research was performed using this NAA concentration in combination with proline. For the evaluation of the proline effect on accumulation of SGs (stevioside and rebaudioside A), phenolic compounds and flavonoids as well antioxidant activity, explants were isolated on MS medium with 2.0 µM NAA and different concentrations of proline (2.0 µM NAA + 2.0 µM PRO, 2.0 µM NAA + 3.0 µM PRO, 2.0 µM NAA + 4.0 µM PRO, and 2.0 µM NAA + 5.0 µM PRO).

Explants were cultivated at 27 ± 2 °C temperature, under illumination of 50 μmol m^−2^ s^−1^ and photoperiod of 16/8 h (day/night) for one month in vitro. The callus was dried at 30 °C for 24 h and used for the analysis.

### 3.3. Callus-Formation Determination 

Callus-formation frequency was determined twice (after one and two week(s)). The percentage of callus formation was calculated using the following formula (explant with cells of callus/total number of explants) × 100%):

$$\mathrm{Callus}\;\mathrm{formation}\;\left(\%\right)=\frac{\mathrm{number}\;\mathrm{of}\;\mathrm{explant}\;\mathrm{with}\;\mathrm{callus}}{\mathrm{total}\;\mathrm{number}\;\mathrm{of}\;\mathrm{explants}}\times 100$$

### 3.4. Callus-Mass Determination

Callus-mass (mg) increase was determined after one and two month(s).

### 3.5. SG Extraction 

The callus was dried at 30 °C for 24 h, powdered using a batch mill with disposable grinding chamber (Tube-Mill control, IKA, Staufen, Germany), and 0.2 g of powder was mixed with 10 mL of deionized water. The extraction was carried out in triplicate by sonication for 60 min at 25 °C. The mixture was centrifuged at 16,000× *g* for 10 min (at room temperature), and the supernatant was collected and kept at −20 °C until analysis by HPLC. The extraction was carried out in triplicate.

### 3.6. HPLC SG Analysis

SGs (steviol glycosides stevioside and rebaudioside A) were separated and quantified using high-performance liquid chromatography (HPLC) by the modified method [53]. An Agilent 1200 series HPLC system (Agilent Technologies Inc., Santa Clara, CA, USA) with a diode array detector was used. Samples were filtered through a syringe filter with a PVDF membrane (pore diameter, 0.22 µm) and separated on a reversed phase column (Purospher STAR RP-18e 5 µm Hibar 2 × 250 mm, Merck, Germany) with a precolumn. Injection volume was 10 µL at 70 °C column temperature. Isocratic elution with a mobile phase consisting of 70% deionized water acidified with HCl to pH 2.75 and 30% acetonitrile was used for separation, with an additional washing step with 50% acetonitrile at a flow rate of 0.25 mL min^−1^. Stevioside and rebaudioside A were detected at the wavelength of 210 nm. Identification of stevioside and rebaudioside A in the samples was done by means of retention time and UV spectra. Calibration was done by plotting the peak area responses against the concentration values in the concentration range from 1 to 1000 µg mL with linear dependence for both analytes. Each analysis was repeated three times, and the mean value was used. Working under an isocratic mode, less than 10 min was required to separate the components of interest without affecting resolution.

### 3.7. Extraction Procedure (Phenolics, Flavonoids, Antioxidant Activity)

Dried *Stevia rebaudiana* Bertoni material was powdered using a batch mill with a disposable grinding chamber (Tube-Mill control, IKA-Werke, Staufen, Germany), and 0.2 g of powder (weighed with 0.0001 g precision) was mixed with 10 mL of 70% ethanol solution. Extraction was carried out in triplicate by ultrasonication for 60 min at 25 °C in an EMAG model Emmi-20HC ultrasonic bath (EMAG AG, Mörfelden-Walldorf, Germany). The mixture was centrifuged at 16,000× *g* for 10 min, and the supernatant was collected and kept at −20 °C until analysis.

### 3.8. Total-Phenolic-Content Determination

Total phenolic content was determined using the modified Folin–Ciocalteu method [54]. We mixed 0.2 mL of stevia extract with 1 mL of 0.2 N Folin–Ciocalteu reagent and 0.8 mL 7.5% sodium carbonate solution. After 60 min of incubation in the dark at room temperature, absorbance was measured at 760 nm. Gallic acid was used as a standard, and results were expressed by mg of gallic acid equivalent (GAE) mg g^−1^ of dry mass. This study was performed using a spectrophotometer.

### 3.9. Total-Flavonoid-Content Determination

Total flavonoid content was analyzed by a colorimetric method on the basis of the complexation of phenolic compounds with Al (III) [54]. We mixed 80 µL of stevia extract with 1920 µL of a reagent containing 40% ethanol, 0.7% acetic acid, 0.4% hexamethylenetetramine, and 0.6% aluminum chloride. After 30 min of incubation in the dark at 4 °C, absorbance was measured at 407 nm. Rutin was used as a standard, and results were expressed by mg of rutin equivalent (RUE) mg g^−1^ of dry mass. This study was performed using a spectrophotometer.

### 3.10. Antioxidant-Activity Determination

Antioxidant activity was measured on the basis of the scavenging of the stable 2,2-diphenyl-1-picrylhydrazyl (Sigma–Aldrich, Taufkirchen, Germany) free radical (DPPH) as described by researchers [55], with some modifications. Accordingly, 50 µL of stevia extract were mixed with 1950 µL of a DPPH solution (0.025 mg mL, prepared in acetonitrile:methanol:sodium acetate buffer (100 mM, pH 5.5) (1:1:2)). After 15 min of incubation in the dark at room temperature, absorbance was measured at 515 nm. Rutin was used as a standard, and antioxidant activity was expressed by mg of rutin equivalent (RUE) mg g^−1^ of dry mass. This study was performed using a spectrophotometer.

### 3.11. Statistical Analysis

All experiments were conducted using a completely randomized design with 3 replicates per treatment and 20 explants per each replicate.

Statistical analysis of the experiment data was done with analysis of variance (ANOVA) using software package STATISTICA (Statistica 12; StatSoft, Inc., Tulsa, OK, USA). The mean values of the amount of SGs, phenols, flavonoids, antioxidant activity, and standard error (SE) were calculated on the basis of the number of independent replications. The effect of factors (explant type, auxin, and proline concentration) and their interaction on active compounds were studied by two-way analysis of variance. Tukey’s honestly significant difference (HSD) test was used for multiple comparisons (*p* < 0.05). Principal-component analysis was performed using XLSTAT Software (XLSTAT, 2018, New York, NY, USA) to analyze the association between applied proline concentrations and bioactive compounds, rebaudioside A/stevioside ratio, and antioxidant activity in callus cultures of S. *rebaudiana* Bertoni.

## 4. Conclusions

The study showed that the significantly highest callus-induction frequency and mass increase were obtained from leaf explants in the MS medium supplemented with 2.0 µM NAA. The highest amount of SGs was determined in the callus from the leaf explants. Proline reduced the amount of SGs. The significantly highest ratio of RebA/Stev was obtained in the callus from the stem segments cultured on the medium with 2.0 µM NAA + 5.0 µM PRO. The significantly highest amount of phenolic compounds was obtained in the callus from the leaf explants in the medium supplemented with 2.0 µM NAA + 2.0 µM PRO. Proline significantly reduced the amount of flavonoids in the callus from leaf explants; the significantly highest amount (5.94 mg g^−1^) of flavonoids was obtained in the medium supplemented only with 2.0 µM NAA. The strongest antioxidant activity of the callus from the leaf explants was in the MS medium with 2.0 µM NAA + 5.0 µM PRO. PCA results show that the identified bioactive compounds and antioxidant activity of the cellular compounds of callus from the leaf and stem explants varied depending on the concentration of proline.

## Figures and Tables

**Table 1:** Effect of growth regulators on callus formation frequency from stem and leaf explants. Mean value ± standard error, n = 3.

Treatments	Callus Formation Frequency %	
	After One Week	After Two Weeks
	Callus from Leaf	
**MS (−)**	0.0 ± 0.0	0.0 ± 0.0
1.0 µM NAA	69.4 ± 6.3 b	100.0 ± 0.0 a
2.0 µM NAA	83.3 ± 0.0 a	100.0 ± 0.0 a
3.0 µM NAA	69.4 ± 4.4 b	87.7 ± 0.8 cb
1.0 µM NAA + 1.0 µM BAP	58.3 ± 0.0 bcd	100.0 ± 0.0 a
2.0 µM NAA + 1.0 µM BAP	58.3 ± 0.0 bcd	100.0 ± 0.0 a
3.0 µM NAA + 1.0 µM BAP	58.3 ± 0.0 bcd	100.0 ± 0.0 a
*Average*	*66.17*	*97.95*
	Callus from stem	
MS (−)	0.0 ± 0.0	0.0 ± 0.0
1.0 µM NAA	47.2 ± 6.3 d	80.3 ± 5.2 c
2.0 µM NAA	61.6 ± 0.0 bc	91.0 ± 9.2 ab
3.0 µM NAA	63.8 ± 4.8 b	69.5 ± 0.1 d
1.0 µM NAA + 1.0 µM BAP	59.5 ± 1.3 bcd	79.3 ± 0.5 c
2.0 µM NAA + 1.0 µM BAP	58.3 ± 6.9 bcd	83.2 ± 4.7 cb
3.0 µM NAA + 1.0 µM BAP	49.9 ± 6.2 cd	81.8 ± 2.0 cb
*Average*	*56.72*	*80.85*
*p-*Value (growth regulator x explant type)	<0.00001	n.s

Note: Means not sharing a common letter were significantly different in column (*p* < 0.05); NAA, α –naphthalene acetic acid; BAP, 6-benzylaminopurine.

**Table 2:** Effect of growth regulators on callus-mass increase from stem and leaf explants. Mean value ± standard error, n = 3.

Treatments	Callus Mass Increase mg	
	After one Month	After two Months
	Callus from Leaf	
MS (−)	0.0 ± 0.0	0.0 ± 0.0
1.0 µM NAA	10.0 ± 0.9 d	19.3 ± 1.9 d
2.0 µM NAA	25.7 ± 1.2 a	39.3 ± 0.1 a
3.0 µM NAA	9.4 ± 0.1 d	36.5 ± 1.0 b
1.0 µM NAA + 1.0 µM BAP	16.1 ± 0.3 c	26.5 ± 1.8 c
2.0 µM NAA + 1.0 µM BAP	18.4 ± 1.0 b	29.7 ± 1.9 bc
3.0 µM NAA + 1.0 µM BAP	15.8 ± 0.8 c	32.1 ± 2.2 b
*Average*	*15.90*	*30.5*
	Callus from stem	
MS (−)	0.0 ± 0.0	0.0 ± 0.0
1.0 µM NAA	2.8 ± 0.3 ef	6.3 ± 0.5 f
2.0 µM NAA	3.7 ± 0.3 e	8.3 ± 0.1 e
3.0 µM NAA	0.4 ± 0.05 g	2.5 ± 0.1 g
1.0 µM NAA + 1.0 µM BAP	0.6 ± 0.0 g	5.0 ± 0.8 f
2.0 µM NAA +1.0 µM BAP	1.0 ± 0.3 g	6.7 ± 0.3 f
3.0 µM NAA + 1.0 µM BAP	1.8 ± 0.2 fg	6.3 ± 0.2 f
*Average*	*1.71*	*5.85*
*p-*Value (growth regulator x explant type)	<0.00100	<0.00001

Note: Means not sharing a common letter were significantly different in column (*p* < 0.05); NAA, α –naphthalene acetic acid; BAP, 6-benzylaminopurine.

**Table 3:** Effect of NAA and PRO on accumulation of steviol glycosides and ratio of rebaudioside A/stevioside (RebA/Stev) in callus culture from leaf and stem explants. Mean value ± standard error, n = 3.

Treatments	Stevioside	Rebaudioside A	RebA/Stev Ratio
	mg g^−1^		
	Callus from Leaf		
**2.0 µM NAA (control)**	**0.40 ± 0.05 a**	0.09 ± 0.001 a	0.23 ± 0.02 b
2.0 µM NAA + 2.0 µM PRO	0.20 ± 0.09 c	0.009 ± 0.0001 f	0.04 ± 0.0001 d
2.0 µM NAA + 3.0 µM PRO	0.20 ± 0.05 c	0.05 ± 0.005 b	0.25 ± 0.10 b
2.0 µM NAA + 4.0 µM PRO	0.39 ± 0.01 ab	0.02 ± 0.003 de	0.05 ± 0.05 d
2.0 µM NAA + 5.0 µM PRO	0.30 ± 0.05 b	0.01 ± 0.004 ef	0.03 ± 0.02 d
*Average*	*0.329*	*0.036*	*0.12*
	Callus from stem		
2.0 µM NAA (control)	0.06 ± 0.005 d	0.04 ± 0.003 bc	0.66 ± 0.11 b
2.0 µM NAA + 2.0 µM PRO	0.04 ± 0.003 e	0.03 ± 0.005 cd	0.75 ± 0.07 b
2.0 µM NAA + 3.0 µM PRO	0.04 ± 0.008 e	0.009 ± 0.0001 f	0.22 ± 0.01 c
2.0 µM NAA + 4.0 µM PRO	0.04 ± 0.003 e	0.005 ± 0.0009 f	0.12 ± 0.01 cd
2.0 µM NAA + 5.0 µM PRO	0.04 ± 0.003 e	0.04 ± 0.002 bc	1.00 ± 0.04 a
*Average*	*0.044*	*0.025*	*0.55*
*p-*Value (proline concentration x explant type)	<0.00542	<0.00001	<0.00001

Note: Means not sharing a common letter were significantly different in column (*p* < 0.05); NAA, α–naphthalene acetic acid; PRO, proline.

**Table 4:** Simple Pearson’s correlation of total phenolics with antioxidant activity in *S. rebaudiana* Bertoni cellular compounds of callus from leaf and stem explants in response to different concentrations of proline.

Callus from Leaf.	r^2^	Coefficient of Correlation %	*p-*Value
2.0 µM NAA (control)	0.93	96.80	0.0320
2.0 µM NAA + 2.0 µM PRO	0.47	68.32	n.s^.1^
2.0 µM NAA + 3.0 µM PRO	0.96	98.03	0.0196
2.0 µM NAA + 4.0 µM PRO	0.71	84.47	0.0167
2.0 µM NAA + 5.0 µM PRO	0.89	94.66	0.0146
**Callus from stem**			
2.0 µM NAA (control)	0.96	97.79	0.0020
2.0 µM NAA + 2.0 µM PRO	0.56	75.08	n.s.
2.0 µM NAA + 3.0 µM PRO	0.85	92.50	0.0444
2.0 µM NAA + 4.0 µM PRO	0.91	95.81	0.0419
2.0 µM NAA + 5.0 µM PRO	0.43	65.44	n.s.

n.s., not significant; NAA, α–naphthylene acetic acid; PRO, proline.
